# First person – Alexander Akerberg

**DOI:** 10.1242/dmm.042440

**Published:** 2019-10-01

## Abstract

First Person is a series of interviews with the first authors of a selection of papers published in Disease Models & Mechanisms (DMM), helping early-career researchers promote themselves alongside their papers. Alexander Akerberg is first author on ‘ Deep learning enables automated volumetric assessments of cardiac function in zebrafish’, published in DMM. Alexander is a postdoctoral research fellow in the lab of Caroline E. Burns and C. Geoffrey Burns at Boston Children's Hospital, MA, USA, where he enjoys building new tools while investigating the molecular mechanisms that govern cardiac form and function.


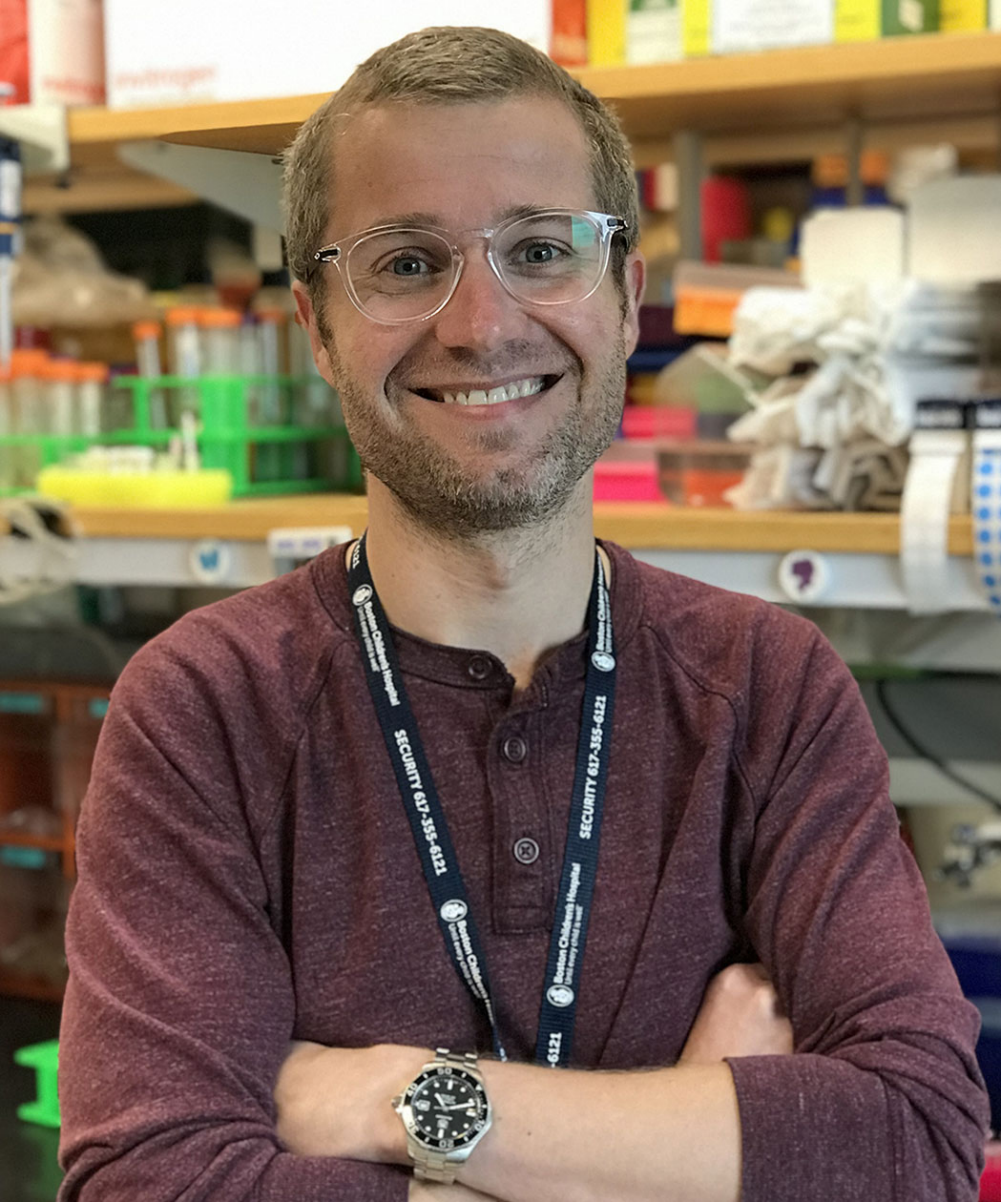


**Alexander Akerberg**

**How would you explain the main findings of your paper to non-scientific family and friends?**

Zebrafish are particularly well-suited for studying heart development and disease; however, the diminutive size of embryonic zebrafish hearts makes it difficult to accurately quantify functional changes that arise in response to an experimental treatment or genetic perturbation. Most researchers rely on a metric called ‘fractional shortening’ to assess cardiac contractility in these animals, which, despite ease of use, produces only a rough approximation of cardiac function and is subject to confounding factors and inaccuracies. We sought to overcome these limitations by creating a novel technique to directly measure cardiac chamber volumes over time, thereby allowing the calculation of more robust and clinically relevant indicators of cardiac function, such as ejection fraction. To accomplish this, we used light-sheet fluorescent microscopy to image beating embryonic zebrafish hearts and trained a deep-learning neural network to extract volumetric data from these images. We then show that our platform, called CFIN, can rapidly and accurately quantify cardiac chamber volumes during both diastole and systole, enabling us to detect chemically induced functional changes that were overlooked by traditional techniques, such as fractional shortening.

**What are the potential implications of these results for your field of research?**

To our knowledge, CFIN currently represents the only platform capable of efficiently making volumetric assessments of cardiac function in zebrafish embryos and, as such, we anticipate it being of great value to the field of cardiovascular research. Additionally, our platform has the potential to be adapted to evaluate systems outside the heart, thereby further expanding its utility.

“To our knowledge, CFIN currently represents the only platform capable of efficiently making volumetric assessments of cardiac function in zebrafish embryos and, as such, we anticipate it being of great value to the field of cardiovascular research.”

**What are the main advantages and drawbacks of the model system you have used as it relates to the disease you are investigating?**

Unlike mice, zebrafish embryos develop outside the mother and are largely transparent, making them a powerful model organism for studying early heart development and disease. Despite molecular and physiological similarities to mammalian hearts, zebrafish have only two cardiac chambers as opposed to four, which can sometimes make direct phenotypic comparisons challenging.

**What has surprised you the most while conducting your research?**

I think what surprised us the most was how well CFIN performed when tasked to label both atrium and ventricle in images that lacked a clear boundary between the two chambers, as is the case when imaging through the atrioventricular canal. Not only did the network correctly segment and classify each chamber in these images, it was also able to appropriately assign mutually exclusive regions within the atrioventricular canal to either ventricle or atrium. What makes this surprising is that this region was deliberately avoided during manual segmentation (i.e. network training) because absolute boundaries between chambers in this region do not exist. In this manner, CFIN actually exceeded our expectations, which was a fascinating and welcomed surprise.

**Figure:**
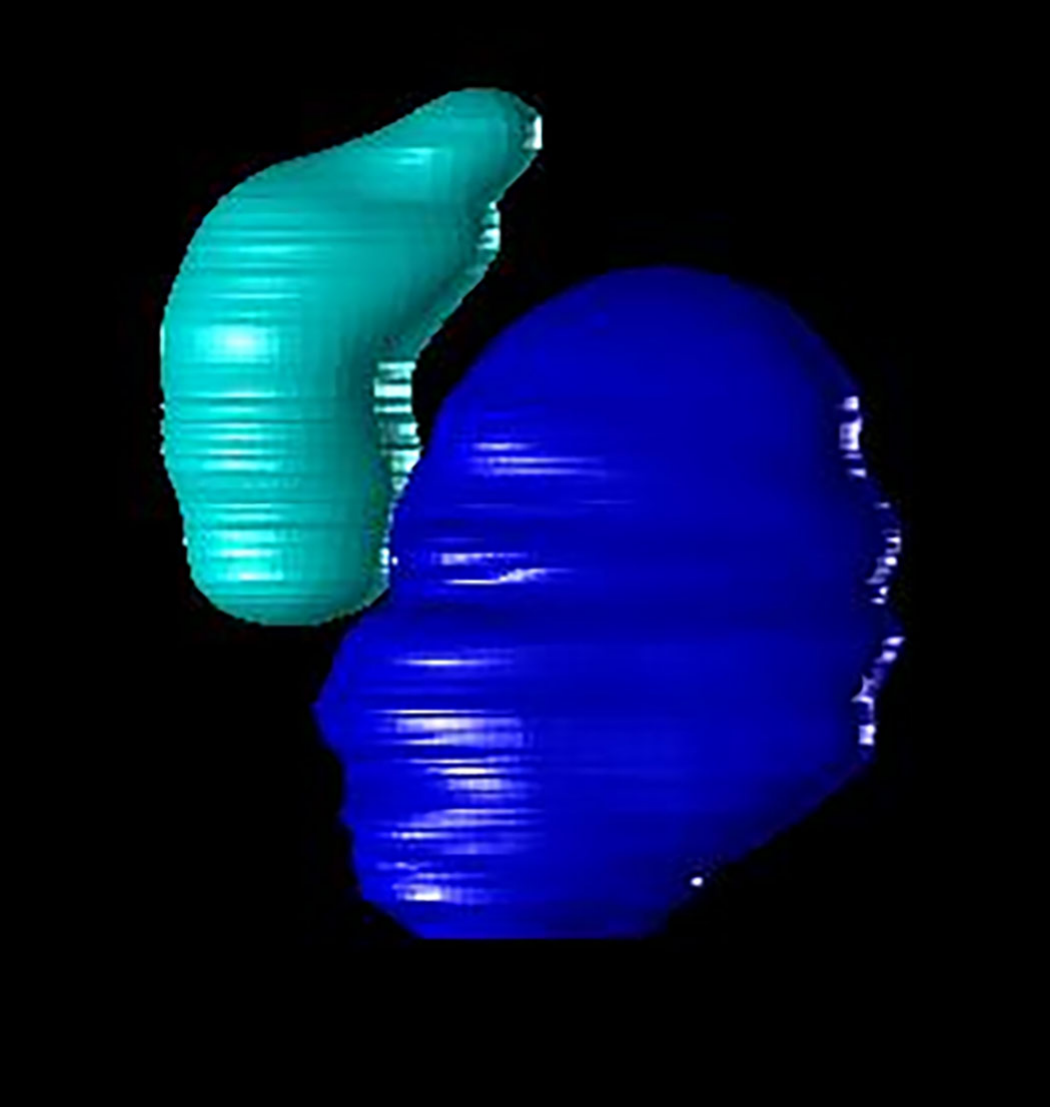
**Volumetric reconstruction of the embryonic heart during ventricular systole (contraction).** Ventricle is shown in cyan and atrium in blue.

“What surprised us the most was how well CFIN performed when tasked to label both atrium and ventricle in images that lacked a clear boundary between the two chambers”

**Describe what you think is the most significant challenge impacting your research at this time and how will this be addressed over the next 10 years?**

Due to an ancestral whole-genome duplication event in the teleost lineage, zebrafish contain dual copies of many genes. This often complicates genetic studies and can make it difficult to extrapolate human relevance. However, with the proliferation of accessible methods for targeted mutagenesis, such as CRISPR-Cas9, researchers over the next 10 years (and beyond) will gradually parse through the zebrafish genome and map instances of genetic redundancy as well as those of functional divergence. Together, these studies will help follow-up investigations better evaluate how findings in zebrafish can advance our understanding of human biology and disease.

**What changes do you think could improve the professional lives of early-career scientists?**

I think having access to more funding opportunities that are specifically designed to help early-career scientists' transition from postdoctoral work into starting an independent lab would be of enormous value. In a broader context, I think it's crucial that we promote scientific literacy among the general population such that more people can appreciate the importance of scientific research and those that dedicate their lives to such pursuits.

“I think it's crucial that we promote scientific literacy among the general population such that more people can appreciate the importance of scientific research and those that dedicate their lives to such pursuits.”

**What's next for you?**

Upon the completion of my postdoctoral training, I intend to start my own lab that utilizes cutting-edge optical, molecular and computational techniques to investigate the RNA-mediated mechanisms that guide cardiac form and function. I am currently utilizing tools like CFIN to investigate the heart-specific roles of RNA-binding proteins during development, which I hope will lay the groundwork for these future endeavors.
